# Infants’ Feeding Habits and Brief Resolved Unexplained Events (BRUEs): A Prospective Observational Study

**DOI:** 10.3390/jcm14061910

**Published:** 2025-03-12

**Authors:** Paolo Quitadamo, Caterina Mosca, Alessandra Verde, Giulio De Marco, Valentina Giorgio, Francesco Valitutti, Pasquale Dolce, Marisa Piccirillo, Melania Evangelisti, Marialuisa Andreozzi, Ludovica Carangelo, Giovanni Di Nardo

**Affiliations:** 1 Pediatric Gastroenterology and Hepatology Unit, Santobono-Pausilipon Children’s Hospital, 80129 Naples, Italy; paoloquitadamo@yahoo.it; 2 Department of Translational Medical Science, Section of Pediatrics, University of Naples Federico II, 80138 Naples, Italy; caterina.mosca@unina.it (C.M.); alessandra.verde@unina.it (A.V.); 3 ASL Napoli 1 Centro, 80121 Naples, Italy; giulio.demarco@aslnapoli1centro.it; 4 Pediatric Unit, Fondazione Policlinico Universitario A. Gemelli IRCCS, 00168 Rome, Italy; valentina.giorgio@policlinicogemelli.it; 5 Pediatric Unit, Department of Maternal and Child Health, Azienda Ospedaliera Universitaria San Giovanni di Dio e Ruggi d’Aragona, 84131 Salerno, Italy; francescovalitutti@unisa.it; 6 Department of Public Health, University of Naples Federico II, 80138 Naples, Italy; pasquale.dolce@unina.it; 7 NESMOS Department, Pediatric Unit, Faculty of Medicine & Psychology, Sapienza—University of Rome, Sant’Andrea University Hospital, 00189 Rome, Italy; marisa.piccirillo@uniroma1.it (M.P.); melania.evangelisti@uniroma1.it (M.E.); 8 Pediatric Unit, San Pio Hospital, 82100 Benevento, Italy; marialuisa.andreozzi@aornsanpio.it; 9 Clinical Pharmacology and Toxicology Unit, Department of Neuroscience and Reproductive and Odontostomatological Sciences, University of Naples Federico II, 80138 Naples, Italy; l.carangelo@santobonopausilipon.it; 10 Clinical and Translational Research Unit, Santobono-Pausilipon Children’s Hospital, 80122 Naples, Italy

**Keywords:** apnea: ALTE, body weight gain, breastfeeding

## Abstract

**Background:** A brief resolved unexplained event (BRUE) is a brief, sudden episode occurring in infants younger than 1 year of age, characterized by some combination of absent, decreased, or irregular breathing, an altered level of responsiveness, color change, and change in muscle tone. Although inappropriate feeding has been suggested as playing a role in the occurrence of BRUEs, only anecdotal reports have been described. The main objective of our study was to objectively evaluate whether overfeeding may represent a risk factor for the occurrence of BRUEs. **Methods:** We enrolled 42 infants aged 0–6 months and admitted for BRUE episodes and the same number of age- and sex-matched healthy infants who served as controls. Data about feeding practices and auxological parameters of each enrolled infant were collected and analyzed, along with clinical data about the pre- and post-natal period. The primary outcome measures were mean daily body weight gain, daily number of feedings, mean volume of feedings, and average daily volume only for bottle-fed infants. **Results:** The mean (±SD) daily body weight gain, the only available and reliable parameter to assess feeding adequacy in both breast- and formula-fed infants, was 41 ± 15 g in infants with BRUEs vs. 35 ± 11 g in healthy infants (95% CI [0.21; 11.8], *p* = 0.042). Moreover, infants with BRUEs were more likely to receive mixed breastfeeding than controls, although this difference did not reach statistical significance (33% vs. 17%, 95 CI [−0.04; 0.37], *p* = 0.131). **Conclusions:** Overfeeding seems to be a risk factor for BRUEs, either through milk inhalation, choking, or GER worsening. Detecting inappropriate feeding practices and providing appropriate education may help prevent the BRUE produced by either scenario.

## 1. Key Messages

### 1.1. What Is Already Known on This Topic

Although overfeeding has been suggested as a risk factor for the occurrence of BRUEs, possibly due to the increased risk of inhalation and choking, only anecdotal reports have been described so far.

### 1.2. What This Study Adds 

Although our study cannot prove a causal relationship between BRUEs and overfeeding, our data suggest that overfeeding is a risk factor for the occurrence of BRUEs.

### 1.3. How This Study Might Affect Research, Practice, or Policy

Avoiding overfeeding may play an essential role in preventing the occurrence of a BRUE episode or, at least, the re-occurrence of BRUEs after the first episode.

## 2. Background

A brief resolved unexplained event (BRUE), previously defined as ALTE (apparent life-threatening event), is defined as a brief, sudden episode occurring in infants younger than 1 year of age, including ≥1 of the following: cyanosis or pallor; absent, decreased, or irregular breathing; a marked change in muscular tone (hyper- or hypotonia); an altered level of responsiveness [1].

Although the definition of ALTEs enabled researchers to establish over time that these events were a separate entity from the sudden infant death syndrome (SIDS), the clinical application of this classification, which describes a constellation of observed, subjective, and nonspecific symptoms, has raised significant challenges for clinicians and parents in the evaluation of and care for these infants [2,3]. ALTEs can create a feeling of uncertainty in both the caregiver and the clinician. Although there are historical and physical examination factors that can determine lower or higher risk, the term ALTE has been replaced with BRUE to advance the quality of care and improve research [1].

The reported incidence of BRUEs ranges from 0.5 to 3 per 1000 live births [4]. Most infants appear to be in clinical well-being when they reach the emergency room. However, they generally undergo a thorough diagnostic work-up to rule out severe medical conditions. The assessment of an infant with a BRUE thus becomes a lengthy and costly process, which can very often be highly bothersome for pediatricians and parents. For many diagnostic tests, the probability that the result is positive is low, which leads to the etiological diagnosis underlying the event being even lower. Gastroesophageal reflux disease, seizures, and lower respiratory tract infections are the most common diagnoses in infants with BRUEs. However, many cases are idiopathic [5].

Although overfeeding has been suggested as a risk factor for the occurrence of BRUEs, possibly due to the increased risk of inhalation and choking, only anecdotal reports have been described [4]. Indeed, a recent clinical study on the relationship between inappropriate feeding and BRUEs highlighted a higher rate of overall inappropriate feeding (over- and under-feeding) and a higher mean weight percentile in infants with BRUEs compared to healthy infants [6]. Difficulty defining appropriate feeding and ascertaining actual feeding practices at the patient’s home are perhaps methodological issues that have impeded further research in this area. Despite these challenges, we decided to explore the association between BRUEs and inappropriate feeding practices toward overfeeding, as they can be modified through parental education at a minimal cost.

The main objective of the present study was to evaluate whether overfeeding may represent a risk factor for the occurrence of BRUEs. The secondary objectives were to assess whether breastfeeding provides a lower likelihood for the occurrence of BRUEs (vs. bottle feeding and mixed breastfeeding) and to investigate the possible cardiac and neurological disorders underlying the BRUE clinical picture.

## 3. Methods

This multicenter prospective observational study was conducted from September 2021 to February 2022 at the pediatric departments of the following hospitals: Santobono-Pausilipon Children’s Hospital in Naples, University of Naples “Federico II” ASL Napoli 1 Centro, A. Gemelli IRCCS Hospital in Rome, San Giovanni di Dio e Ruggi d’Aragona Hospital in Salerno, and Sant’Andrea Hospital in Rome.

Cases were selected among infants aged 0–6 months, admitted for BRUE episodes as defined by the American Academy of Pediatrics (AAP) [1]. Preterm infants (gestational age < 32 weeks) and infants affected by comorbidities, such as respiratory and allergic disorders, were excluded.

For each enrolled infant, data about the peri- and post-natal period, eating habits (breastfeeding or bottle feeding, number of daily feedings, duration of feeding, volume of feeding for formula-fed infants), auxological parameters including weight and recent weight gain, and history of regurgitation were collected. Moreover, the results of clinical, laboratory, and instrumental examinations within the BRUE diagnostic work-up were registered, along with any acute illness at admission and any treatment prescribed at discharge.

Age- and sex-matched healthy infants were recruited at the outpatient clinics of the involved general pediatricians during routine well-child visits and served as controls. Medical records were completed for each patient, and the same clinical, auxological, and nutritional data were gathered for the study cases.

To define and assess overfeeding, we considered the primary outcome of feeding status, the mean daily body weight gain, as the only reliable parameter that could be computed for every infant, regardless of feeding modalities. However, since we did not find any scientific data about the mean daily body weight gain, we defined the sample size according to the total volume of daily feedings. Considering 800 g (±192 g) as the mean amount (±SD) of milk consumed daily by healthy infants aged 0–6 months and assuming that the standard deviation was the same in both groups, a sample size of 42 patients per study group reached a statistical power of 80% to detect a difference of 120 g in daily milk intake between the two groups, using a two-tailed *t*-test [5]. A *p*-value of ≤0.05 was considered statistically significant. Categorical variables were summarized as frequencies and percentages, and comparisons between the two groups were verified using the χ^2^ test. Quantitative variables were summarized as mean and standard deviation, and the Student’s t-test was used to compare the study groups. The variable daily number of feedings was summarized as median and interquartile range (IQR), and the non-parametric Mann–Whitney test was performed for group comparison. The Pearson correlation coefficient was used to analyze the relationship between the “mean daily weight gain” and the “chronological age”. All statistical analyses were performed using the R software 4 for statistical computing.

The study was approved by the “Cardarelli-Santobono” Independent Ethics Committee and was conducted in accordance with the Declaration of Helsinki and Guidelines for Good Clinical Practice.

## 4. Results

Over the study period, we enrolled 42 infants with BRUEs (male/female: 22/20; mean age ± standard deviation: 75 ± 47 days; age range: 24–175 days) and 42 sex- and age-matched healthy infants who served as controls (male/female: 21/21; mean age ± standard deviation: 77 ± 47 days; age range: 28–180 days). BRUE episodes were characterized mainly by apnea, hypotonic rather than hypertonic muscle tone, cyanosis rather than skin pallor, choking, and gagging. According to the AAP classification, 21/42 infants (50%) could be included in the lower-risk and 21/42 (50%) in the higher-risk patient group.

All cases underwent a diagnostic evaluation to exclude alternative diagnoses. The diagnostic work-up variably included clinical observation with continuous vital parameter monitoring, cardiological consultation with electrocardiography and echocardiography, neurological consultation with or without electroencephalography (according to the clinical presentation of the BRUE episode), gastroenterological consultation with possible esophageal pH-impedance monitoring, and blood tests (complete blood cell count, biochemical parameters, C-reactive protein, and arterial blood gas). Although no significant cardiologic or neurologic disorders were detected, two infants started neurological follow-ups due to minor electroencephalographic alterations.

There were no significant differences between the BRUE and control groups concerning birth weight (3000 g versus 3142 g; 95% CI (−372; 88], *p* = 0.223). However, BRUE infants had a mean gestational age ± SD that was significantly lower than that of healthy infants (37.6 ± 2.1 vs. 38.6 ± 1.4; 95% CI (−1.80; 0.25], *p* = 0.010).

Feeding practices and study groups were statistically independent (Table 1). Thus, no significant differences in the prevalence of breastfeeding, bottle feeding, and mixed feeding were observed between the two study groups. Indeed, infants with BRUEs were more likely to have received mixed breastfeeding than controls, although this difference did not reach statistical significance (33% vs. 17%, 95 CI [−0.04; 0.37], *p* = 0.131). The mean volume of feedings of bottle-fed infants was 130 ± 47 mL in the BRUE group and 137 ± 30 in the control group (95% CI [−28; 14], *p* = 0.512), while the median daily number of feedings was 7.45(1.4) vs. 6(1), respectively (*p* = 0.001). Therefore, the average daily volume of feedings was 868 ± 260 in the BRUE group and 775.6 ± 235 in the control group (*p* = 0.176).

**Figure 1:**
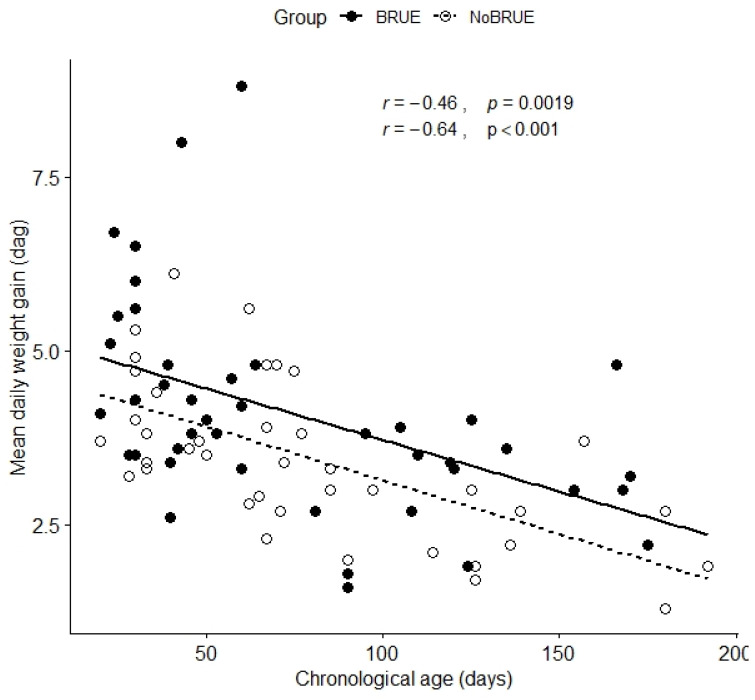
Scatter plot of mean body weight gain and chronological age. Black and white circles represent the BRUE and control groups, respectively.

The mean daily body weight gain was 41 ± 15 g in infants with BRUEs vs. 35 ± 11 g in healthy infants (95% CI [0.21; 11.8], *p* = 0.042) (Figure 1). Finally, parents of infants with BRUEs reported a clinical history of regurgitation (at least three daily episodes for at least 3 days per week) more frequently than those of healthy infants (24/42 vs. 14/42; *p* = 0.047).

## 5. Discussion

Our main finding is that infants with BRUEs had a significantly higher mean daily body weight gain over the previous weeks than healthy age- and sex-matched infants.

Reliable parameters assessing infants’ overfeeding need to be included, mainly because there are no exact recommendations from the World Health Organization about the appropriate volume per feeding or the total volume of daily feedings for healthy infants. Moreover, even if they did exist, these data would not be achievable for breastfed infants. Without cut-off values, we could not classify infants according to their actual feeding status as overfed and not overfed. We could only analyze continuous variables, among which we considered the mean daily body weight gain to be the only objective and reliable parameter of feeding adequacy.

The difference in daily body weight gain reached statistical significance within the two study groups. Indeed, from a clinical perspective, 6 g daily (on an average of 30–40 g) may be noticed. We can, therefore, infer that overfeeding may represent a risk factor for the occurrence of BRUEs. This association suggests the possible role of the relationship between overfeeding and gastroesophageal reflux (GER). GER has frequently been cited as a cause of BRUEs/ALTEs, even if a causal relationship has not been verified [7]. Previous findings suggest that the underlying mechanism through which inappropriate feeding predisposes an infant to an ALTE may not be an exacerbation of GER but rather a dysfunctional coordination between respiration and swallowing. However, in our study sample, we found a higher prevalence of regurgitation in infants with BRUEs, thus supporting the hypothesis of GER as the possible link between overfeeding and BRUE symptoms [7].

To our knowledge, no previous study has examined the association between BRUEs and overfeeding. The only report addressing the role of feeding adequacy in the occurrence of BRUEs was published by Kojima et al. in 2017 [6]. The authors compared the feeding behaviors of infants who have had an ALTE with age- and sex-matched controls. The ALTE group had a significantly lower prevalence of appropriate feeding than the control group. The infants who were inappropriately fed were more often overfed rather than underfed. However, the prevalence of overfeeding was similar in both the ALTE and control groups.

We did not report statistically significant differences in feeding practices (breastfeeding vs. bottle feeding) between the two study groups. However, we observed a trend toward statistical significance for mixed breastfeeding, which was more commonly reported within infants with BRUEs than controls. Even if not conclusive, in our opinion, these data agree with the higher rate of overfeeding in the BRUE group since it is not uncommon for mixed breastfeeding to facilitate the intake of a greater volume of milk compared to exclusive breastfeeding or exclusive formula feeding.

Infants admitted for BRUEs had a higher daily number of feedings than healthy infants in the control group. At the same time, no differences were reported in the mean amount of such feedings or the total daily feeding amount. Finally, BRUE infants had a mean gestational age significantly lower than healthy infants.

One of the strengths of our study is the use of objective and reliable criteria to determine the feeding status, both in breast- and formula-fed infants. We excluded pre-term infants, as their body sizes are smaller in relation to term infants of the same chronological age. We also ensured that the BRUE and control groups had similar ages and body sizes in relation to their chronological ages at the time of presentation.

Although our study cannot prove a causal relationship between BRUEs and overfeeding, we may speculate that overfeeding (measured by daily body weight gain) represents a risk factor for BRUEs, either through milk inhalation and choking or GER worsening. Detecting inappropriate feeding practices and providing appropriate education to correct them may prevent the occurrence of a BRUE produced by either scenario. Therefore, primary care pediatricians must perform thorough feeding evaluations during routine well-child visits to detect at-risk infants and prevent BRUEs.

## 6. Conclusions

In conclusion, we found a significantly higher recent body weight gain in infants admitted for BRUEs, along with more frequent mixed breastfeeding and more frequently reported GER episodes. Education regarding feeding practices and adequacy is essential to general pediatric practice, and our study emphasizes its importance. Multiple studies have suggested that a routine work-up is unnecessary when evaluating an infant presenting with a BRUE [8,9,10]. As for other medical conditions, a detailed clinical history and a thorough physical examination are the most important and sometimes crucial tools. Avoiding overfeeding may play an essential role in preventing the occurrence of a BRUE episode or, at least, the re-occurrence of BRUEs after the first episode.

## Figures and Tables

**Table 1:** Comparison of feeding practices between BRUE group and control group.

	BRUE Group (n = 42)	Control Group (n = 42)	*p* Value
Breastfeeding, n (%)	13 (31%)	15 (35.7%)	0.6
Formula feeding, n (%)	15 (35.7%)	20 (47.6%)	0.7
Mixed feeding, n (%)	14 (33.3%)	7 (16.7%)	0.2

BRUE: brief resolved unexplained event.

## Data Availability

The datasets used and analyzed during the current study are available from the corresponding author upon reasonable request.

## Abbreviations

ALTE apparent life-threatening event, BRUE brief resolved unexplained event, SIDS sudden infant death syndrome.

## References

[1] Tieder J.S., Bonkowsky J.L., Etzel R.A., Franklin W.H., Gremse D.A., Herman B., Katz E.S., Krilov L.R., Norlin C., Percelay J. (2016). Brief Resolved Unexplained Events (Formerly Apparent Life-Threatening Events) and Evaluation of Lower-Risk Infants. Pediatrics.

[2] Tieder J.S., Altman R.L., Bonkowsky J.L., Brand D.A., Claudius I., Cunningham D.J., DeWolfe C., Percelay J.M., Pitetti R.D., Smith M.B.H. (2013). Management of apparent life-threatening events in infants: A systematic review. J. Pediatr..

[3] Merritt J.L., Quinonez R.A., Bonkowsky J.L., Franklin W.H., Gremse D.A., Herman B.E., Jenny C., Katz E.S., Krilov L.R., Norlin C. (2019). A Framework for Evaluating the Higher-Risk Infant After a Brief Resolved Unexplained Event. Pediatrics.

[4] Semmekrot B.A., Van Sleuwen B.E., Engelberts A.C., Joosten K.F.M., Mulder J.C., Liem K.D., Pereira R.R., Bijlmer R.P.G.M., L’Hoir M.P. (2010). Surveillance study of apparent life-threatening events (ALTE) in the Netherlands. Eur. J. Pediatr..

[5] Elzouki A.Y., Harfi H.A., Stapleton F., Stapleton F.B., Oh W., Whitley R.J. (2012). Textbook of Clinical Pediatrics.

[6] Kojima K., Mckinley K., Donohue P., Sigal Y. (2017). The high prevalence of inappropriate feeding among infants presenting with an apparent life-threatening event. Turk. J. Pediatr..

[7] Tolia V., Vandenplas Y. (2009). Systematic review: The extraoesophageal symptoms of gastro-oesophageal reflux disease in children. Aliment. Pharmacol. Ther..

[8] Zenzeri L., Quitadamo P., Tambucci R., Ummarino D., Poziello A., Miele E., Staiano A. (2017). Role of non-acid gastro-esophageal reflux in children with respiratory symptoms. Pediatr. Pulmonol..

[9] Quitadamo P., Tambucci R., Mancini V., Cristofori F., Baldassarre M., Pensabene L., Francavilla R., Di Nardo G., Caldaro T., Rossi P. (2019). Esophageal pH-impedance monitoring in children: Position paper on indications, methodology and interpretation by the SIGENP working group. Dig. Liver Dis..

[10] Macchini F., Morandi A., Cognizzoli P., Farris G., Gentilino V., Zanini A., Leva E. (2017). Acid Gastroesophageal Reflux Disease and Apparent Life-Threatening Events: Simultaneous pH-metry and Cardiorespiratory Monitoring. Pediatr. Neonatol..

